# IPSC derived cardiac fibroblasts of DMD patients show compromised actin microfilaments, metabolic shift and pro-fibrotic phenotype

**DOI:** 10.1186/s13062-023-00398-2

**Published:** 2023-07-27

**Authors:** Salwa Soussi, Lesia Savchenko, Davide Rovina, Jason S. Iacovoni, Andrea Gottinger, Maxime Vialettes, Josè-Manuel Pioner, Andrea Farini, Sara Mallia, Martina Rabino, Giulio Pompilio, Angelo Parini, Olivier Lairez, Aoife Gowran, Nathalie Pizzinat

**Affiliations:** 1 National Institute of Health and Medical Research (INSERM), I2MC, U1297, Toulouse, France; 2 Unit of Vascular Biology and Regenerative Medicine, Centro Cardiologico Monzino IRCCS, Milan, Italy; 3 National Institute of Health and Medical Research (INSERM) U1297 I2MC, Bioinformatic Core Facility, I2MC, Toulouse, France; 4 Department of Biomedical, Surgical and Dental Sciences, Università Degli Studi di Milano, Milan, Italy; 5 Department of Biology, University of Florence, Florence, Italy; 6 Neurology Unit, Fondazione IRCCS Ca’ Granda Ospedale Maggiore Policlinico, Milan, Italy; 7 University Toulouse III, 118 route de Narbonne, 31062 Toulouse, CEDEX 9, Toulouse, France

**Keywords:** Duchenne, Human induced pluripotent stem cell, Fibroblasts, Mitochondrial oxidation, Actin microfilaments

## Abstract

Duchenne muscular dystrophy (DMD) is a severe form of muscular dystrophy caused by mutations in the dystrophin gene. We characterized which isoforms of dystrophin were expressed by human induced pluripotent stem cell (hiPSC)-derived cardiac fibroblasts obtained from control and DMD patients. Distinct dystrophin isoforms were observed; however, highest molecular weight isoform was absent in DMD patients carrying exon deletions or mutations in the dystrophin gene. The loss of the full-length dystrophin isoform in hiPSC-derived cardiac fibroblasts from DMD patients resulted in deficient formation of actin microfilaments and a metabolic switch from mitochondrial oxidation to glycolysis. The DMD hiPSC-derived cardiac fibroblasts exhibited a dysregulated mitochondria network and reduced mitochondrial respiration, with enhanced compensatory glycolysis to sustain cellular ATP production. This metabolic remodeling was associated with an exacerbated myofibroblast phenotype and increased fibroblast activation in response to pro fibrotic challenges. As cardiac fibrosis is a critical pathological feature of the DMD heart, the myofibroblast phenotype induced by the absence of dystrophin may contribute to deterioration in cardiac function. Our study highlights the relationship between cytoskeletal dynamics, metabolism of the cell and myofibroblast differentiation and provides a new mechanism by which inactivation of dystrophin in non-cardiomyocyte cells may increase the severity of cardiopathy.

**Supplementary Information:**

The online version contains supplementary material available at 10.1186/s13062-023-00398-2.

## Introduction

Duchenne muscular dystrophy (DMD) is the most common form of muscular dystrophy caused by mutations in the DMD gene on the X chromosome (Xp21.1-Xp22), [1]. The different mutations observed in Duchenne patients (including exon deletions and duplications, as well as point mutations) that disrupt the translational reading frame result in the lack of the full-length form of Dystrophin, a component of the dystrophin glycoprotein complex (DGC). Dystrophin links the cytoskeleton and the extracellular matrix to help maintain muscle membrane integrity, in part by dissipating the forces of muscle contraction into the extracellular matrix. Thus, deficiencies in dystrophin result in chronic muscle damage, dysregulation of repair and progressive skeletal muscle weakness [2]. Cardiac muscle is also affected by dystrophin loss and often leads to dilated cardiomyopathies [3, 4].Typically, cardiomyopathy begins in the first decade of life and evolves with progressive replacement of the myocardium by fibrous and fatty connective tissue resulting in end-stage dilated cardiomyopathy, which is a major cause of death in DMD patients [5]. Several mechanical disturbances have been observed in the DMD heart among them; increased heart rate variability, Ca^2+^ homeostasis dysregulation, tissue remodeling with fibro-fatty accumulation and cardiomyocyte death [6]. The dying cardiac cells trigger an inflammatory response that elicits immune cell infiltration and promotes fibrotic scar tissue, which impairs the compliance of the heart and worsens cardiac dysfunction. There may also be additional mechanisms by which dystrophin mutation engenders ventricular fibrosis that have yet to be uncovered.

Skeletal and cardiac myocytes expressed mainly the full-length (427 kDa) dystrophin isoform of the *DMD* gene that comprises 79 exons. The full-length protein consists of at least four main regions; an N-terminal domain plays an essential role in binding actin (ABD1), a central rod domain that harbors a second actin binding domain (ABD2), a cysteine-rich domain, which mainly interacts with the transmembrane protein dystroglycan and therefore indirectly with other DGC membrane proteins, followed by the C-terminal region [7, 8]. In addition, five smaller dystrophin isoforms, named according to their molecular weight (Dp260, Dp140, Dp116, Dp71 and Dp40), can be generated by the differential use of internal promoters. Furthermore, alternative splicing of specific exons, exon junctions or inclusion of intronic sequences can give rise to splice variants with modified carboxy termini [2]. Depending on the domain composition of the various isoforms, they have partially retained the capacity to bind to different proteins of the dystroglycan complex [9]. Although the expression of Dp71 is ubiquitous in cells, the existence of splicing variants has been particularly documented in retina and brain [10–12]. In the heart, dystrophin is abundantly expressed in myocytes, and a large body of work has been devoted to studying the consequences of dystrophin mutation, whereas non-muscle cells have been little studied until now. In this study, we revealed the expression of dystrophin isoforms in hiPSC-derived cardiac fibroblasts. The loss of dystrophin in DMD patients modified actin cytoskeleton, induced a metabolic remodeling, exacerbated the myofibroblast phenotype and worsened fibroblast activation in response to pro fibrotic challenges. Our study highlights the relationship between cytoskeletal dynamics, metabolism of the cell and myofibroblast differentiation. Our work contributes to the understanding of the pathogenic mechanisms that occur in dystrophic cardiopathy.

## Material and methods

### Cell culture and treatments

The isolation of the human cells and the subsequent reprogramming into iPSC lines was performed in conformation with the Declaration of Helsinki. iPS cells from 3 DMD patients and 3 healthy donors were obtained from Centro Cardiologico Monzino, Milan Italy, as previously described [13–15]. The three Duchenne patients carried different *DMD* mutations: two exons deletions and a point mutation. In particular, DMD1 had a deletion of exons 49 and 50 [13], while DMD4 carried a bigger deletion from exon 45 to exon 50 [15]. DMD5 carried a point mutation c.6913–1 G > A; this mutation causes the deletion of a nucleotide in the mature transcript with a shift in the reading frame that causes a premature stop codon.

### hiPSC-EPI differentiation

Cell reprogramming into iPSC lines was previously described by Rovina et al. [15]. Briefly, iPSCs were seeded in Matrigel (Corning, 356,230) coated 6 well plates in StemFlex medium (ThermoFisher, A3349401) supplemented with RevitaCell (ThermoFisher, A2644501). On Day 0, medium was replaced with BPEL medium and cells were treated with 6 mM CHIR99021 (Bertin bioreagent, 13,122), 100 µg/ml Activin A (MiltenyiBiotec, 130–115-008), and 100 µg/ml BMP4 (R&D Systems, 5020-BP). On Day 3, cells were treated with 10 mM XAV939 (R&D Systems,3748), 100 µg/ml BMP4 and 2 mM Retinoic acid (Sigma Aldrich, R2625). Medium is changed on Day6 with BPEL + 30 ng/ml BMP4 + 1 µM retinoic acid. After 3 days, cells were replated on fibronectin coated plates with BPEL medium supplemented with 20 mM SB 431542 (R&D Systems,161).

### hiPSC-fibs differentiation

EPI-iPSC cells were seeded in vitronectin (ThermoFisher, A14700) coated 6 well plates in BPEL medium supplemented with 20 µg/ml FGF-2 (Peprotech, AF-100-18B). Medium was changed every two days for 10 days. At Day10, medium is switched to FGM3 (Promocell, C-39350) and changed every other day. Cells were used between passages 0 and 3. Profibrotic stimulations were performed by incubating iPSC derived cardiac fibroblasts with 10 ng/ml TGF beta or 100 ng/ml angiotensin II for 24 h in FGM3 containing 0.5% SVF.

### RNA sequencing and bioinformatics

RNA was isolated using ReliaPrep RNA Cell Miniprep System (Promega). Samples quantity and quality were evaluated on a Fragment analyzer (Advanced analytical). Libraries were generated with TruSeq stranded mRNA Illumina and quantified on a Fragment analyzer by the GeT-PlaGe Genotoul Toulouse-France.

Sequencing was performed by Integragen (France) via an Illumina NovaSeq™6000 S2 high throughput sequencer resulting in 30 to 38 million paired end reads (2 × 100 bp) per sample. Reads were cleaned with HTStream (v1.3.2) [https://github.com/s4hts/HTStream] and aligned with Salmon (v1.9.0) ([16] using the gcBias option, against the Gencode v40 human transcript database [17]).

The rest of the analysis was performed in R (v4.2.3) [R Core Team (2021). R: A language and environment for statistical computing. R Foundation for Statistical Computing, Vienna, Austria.[https://www.R-project.org/]. Tximport (v1.26.1) [18] was used to load in the Salmon mapping results. Functions from edgeR (v3.40.2) [19] were used to filter genes by expression and calculate normalization factors. limma (v3.54.2) [20]was used for the voom With Quality Weights function and then to perform cyclic Loess normalization with the “affy” method. Then we employed sPLS-DA from the mixOmics (v6.22.0) to select genes that we able to select genes differential with respect to the DMD and CON group factor. Figures were created with mixOmics plotIndiv, a custom ggplot2 (v3.4.2) [H. Wickham. ggplot2: Elegant Graphics for Data Analysis. Springer-Verlag New York, 2016] adaptation of mixOmics’ plotVar and pheatmap (v1.0.12) [https://cran.r-project.org/package=pheatmap].

Pathway enrichment was performed with EnrichR [21] using the KEGG 2021 Human database [22] and HumanCyc 2016 database [23].

RNA-Seq data analysis was done by bioinformatic platform in Toulouse, functional annotation clustering and for up-regulated and down-regulated genes with a fold change of ≥ 2 and enrichment score of ≥ 1.

### Reverse transcription and real time PCR

Total RNA was purified using ReliaPrep RNA Cell Miniprep System (Promega) according to the manufacturer’s instructions. RNA was quantified with NanoDrop (ND 2000, Thermofisher). The same RNA was retrotranscribed with the Applied biosystems Synthesis Kit (ThermoFisher Scientific, 4,368,814). Real time qPCR reactions were performed using TB Green Premix Ex Taq II (Takara) on a QuantStudio5 Real-Time PCR System (ThermoFisher) instrument. Gene expression levels were normalized to GAPDH and 36B4 housekeeping genes. Results were analyzed using the ΔΔCT method.

For mitochondrial DNA quantification, genomic DNA was purified on QIAamp DNA micro isolation kit (QIAGEN) following the manufacturer’s instructions. ND1 and COX1 genes expression was normalized to nuclear DNA B2M using the ΔCT method.

### Seahorse analysis

hiPSC-fibs were seeded into vitronectin coated Seahorse 24 assay plates at a density of 80 000cells/well. Oxygen consumption rate (OCR) and extracellular acidification rate (ECAR) were assessed using a standard mitochondrial and glycolysis stress on the Seahorse Bioscience XF-24 analyzer (Agilent technologies). On the day of metabolic flux analysis, cells were washed with Seahorse XF base medium pH7.4 (Agilent technologies). The culture medium was replaced with 500 µl of Seahorse medium supplemented with 10 mM glucose, 1 mM sodium pyruvate and 2 mM glutamine and incubated 1 h in a CO2-free incubator at 37 °C. For XF glycolysis stress test, cells were incubated in DMEM with 2 mM glutamine. For the XF mito stress, inhibitors of the mitochondrial electron transport chain (oligomycin 1 µM, carbonyl cyanide 4-trifluoromethoxy phenylhydrazone (FCCP) 4 µM, rotenone and antimycin 1 µM) were sequentially injected to assess the OCR and the respiratory parameters. For XF glycolysis stress, glucose 10 mM, oligomycin 1 µM and 2-deoxy-D-glucose (2-DG) were injected to evaluate the ECAR and the glycolysis parameters. OCR and ECAR were automatically calculated by the Seahorse XF-24 software.

### Cellular ATP levels

Intracellular ATP levels were measured using the Cell Titer-Glo 2.0 cell viability assay (Promega). Briefly, 4 × 10^4^ cells were loaded into each well in a 96well plate. To evaluate ATP generated from the oxidative phosphorylation or glycolysis, cells were treated with sodium iodoacetate 100 µM, oligomycin 1.6 µM antimycin A 100 µM (OAA) or PBS and incubated 1 h at 37 °C. 100 µl of Cell Titer Glo was added to each well and luminescence was measured using Infinite F500 reader (Tecan).

### Intracellular lactate levels

Intracellular L-lactate was measured using Lactate colorimetric assay kit (Sigma-Aldrich) according to the manufacturer’s instructions. 1,6 × 10^6^ cells were loaded per well in a 96well plate and mixed with master reaction containing enzyme mix, probe and buffer. After 30 min incubation, the colorimetric intensity was measured at 570 nm on Infinite F200 Pro (Tecan) (Table 1).

**Table 1:** Oligonucleotides used for qPCR

Target	Sequence
36B4 F	CAGATCACGTCATCGCACAAC
36B4 R	AAAAGGAGGTCTTCTCGGGC
α-SMA F	GGAGCAGCCCAGCCAAGC
α-SMA R	AGAGCCCAGAGCCATTGTCAC
COL1A F	GAGGGCCAAGACGAAGACATC
COL1A R	CAGATCACGTCATCGCACAAC
GAPDH F	AAGGTCGGAGTCAACGGATTT
GAPDH R	ATGAAGGGGTCATTGATGGCA
GLUT1 F	GAGGGCCAAGACGAAGACATC
GLUT1 R	CAGATCACGTCATCGCACAAC
TNC F	CAACCATCACTGCCAAGTTCACAA
TNC R	GGGGGTCGCCAGGTAAGGAG

### Western blot analysis

Whole cell lysates were prepared using RIPA lysis buffer (Cell Signaling, 9806) containing protease and phosphatase inhibitor cocktail (Thermofisher scientific,78,420). Concentration of protein lysates were calculated using the BCA protein assay (ThermoFisher, 23,225). 10-50 µg were loaded onto a 12% polyacrylamide gel and subjected to gel electrophoresis. The resolved proteins were transferred with the aid of a Trans-Blot Turbo Transfer System (Bio-Rad) according to the manufacturer’s recommendation (1A, 25 V, 30 min). For dystrophin, resolved proteins were transferred into 0.2 µm nitrocellulose membrane using wet transfer overnight at 4 °C at a constant current of 90 mA.The PVDF membrane was blocked in Tris-Buffered Saline 0.1% Tween TBST containing 5% Bovine Serum Albumin BSA (Sigma) and washed with TBST. The membranes were then incubated with the appropriate antibodies (Table 2). After an overnight incubation period at 4 °C, the membranes were washed in TBST and incubated with appropriate secondary antibody conjugated to horseradish peroxidase for 1h30 at room temperature. Proteins were detected using Clarity western ECL Substrate (Bio-Rad) with the aid of ChemiDoc MP Imaging System (Bio-Rad).

**Table 2:** Antibodies used for western blot and immunofluorescence

Target	Provider	Ref
Collagen I	Cell Signaling Technology	#72026
Dystrophin (Mandra1)	Santa Cruz Biotechnology	sc-73592
Dystrophin (7A10)	Santa Cruz Biotechnology	sc-33697
Dystrophin	Abnova	630-170
Dystrophin	Thermofisher	PA5-16734
DRP1	Abcam	ab184247
HK1	Santa Cruz Biotechnology	sc-46695
HK2	Santa Cruz Biotechnology	sc-374091
HSP60	Cell Signaling Technology	#12165
LDHA	Santa Cruz Biotechnology	sc-137243
LDHB	Santa Cruz Biotechnology	sc-100775
MFN2	Abcam	ab56889
Oxphos	Invitrogen	45-8099
PDHE1a	Santa Cruz Biotechnology	sc-377092
Phospho PDHE1a	Cell Signaling Technology	#31866
PKLR	Santa Cruz Biotechnology	sc-133222
RhoGDi	Santa Cruz Biotechnology	sc-365190
VDAC1	Santa Cruz Biotechnology	sc-390996
α-SMA	Sigma	A5228
β-Tubulin	Cell Signaling Technology	#2146
γ-Actin	Cell Signaling Technology	sc-65638
β -Actin	Santa Cruz Biotechnology	sc-47778

### Flow cytometry

Phenotypic analysis of hiPSC-fibs was performed using multicolor flow cytometry. Briefly, nonspecific binding of antibodies by Fc receptors was blocked by incubating the cells with mouse serum diluted in FACS buffer (PBS 4% FBS and 2 mM EDTA). Cell surface markers were then stained with antibodies conjugated with fluorochromes (Table 3). Viability was assessed by incubation with Live-Dead Yellow (Invitrogen). The following isotype controls mouse IgG1k BV421, mouse IgG1k PE, mouse IgG1k FITC and mouse IgG1k APC were used for compensation setups. Samples were acquired on LSR Fortessa (BD Bioscience). Analysis of flow cytometric data was performed using FlowJo (TreeStar).

**Table 3:** Antibodies used for flow cytometry

Fluorochrome	Target	Clone	Provider	Ref
BV421	CD90	5E10	Biolegend	328121
PE	CD140a (PDGFRα)	16A1	Biolegend	323505
AF488	CD29	TS2/16	Biolegend	303015
APC	CD105	43A3	Biolegend	323208
APC Vio700	CD45	REA747	Miltenyi	130-110-635
PerCp cy5.5	CD31	WM59	Biolegend	303131

### Immunofluorescence staining

Cultured iPSC-CF on vitronectin coated glasses were fixed in paraformaldehyde 4% and permeabilized with Triton 0.1% (Sigma). The cells were blocked for 1h30 and incubated during 1 h with primary antibodies (Table 3) at room temperature, then secondary antibody for 1h30. Subsequently, cells were incubated with Phalloidin (1:400, A12381; Thermo Fisher Scientific) to stain F-actin. After washing steps, cells were marked with DAPI and slides were mounted using fluoromount mounting medium (Invitrogen). Images were taken with a LSM900 Confocal laser scanning microscope using the × 63 oil immersion objective lens. Immunofluorescence quantifications were made on 5–6 images for each condition. Phalloidin and collagen quantification was performed using the mean fluorescence intensity (MFI) on Fiji. Mitochondrial network analysis quantification was performed using MiNA (Mitochondrial Network Analysis) workflow on Fiji, analyzing the ‘’networks’’ and ‘’mitochondrial footprint’’.

### Statistics

Data were obtained from three independent DMD patients and healthy donors. Results are expressed as means ± standard errors of the mean (SEM). Statistical analysis was performed using GraphPad Prism version 9. Statistical difference was analyzed by the student’s T test when comparing two groups and a one-way ANOVA followed by the Tukey posttest for multiple comparisons. Values of *P* < 0.05 were considered statistically significant.

## Results

### The 427 kDa of dystrophin isoform is expressed in healthy human iPS-derived cardiac fibroblasts (control hiPSC-fibs) and absent in hiPSC-fibs isolated from DMD patients

We explored isoforms of dystrophin expressed by hiPSC-derived cardiac fibroblasts (hiPSC-fibs) obtained from control and DMD patients with out of frame deletions of exons (DMD1 and DMD4) and a patient who has a spontaneous point mutation in intron 47 (DMD5) that leads to a frameshift and the formation of a premature stop signal [13–15].

**Fig. 1:**
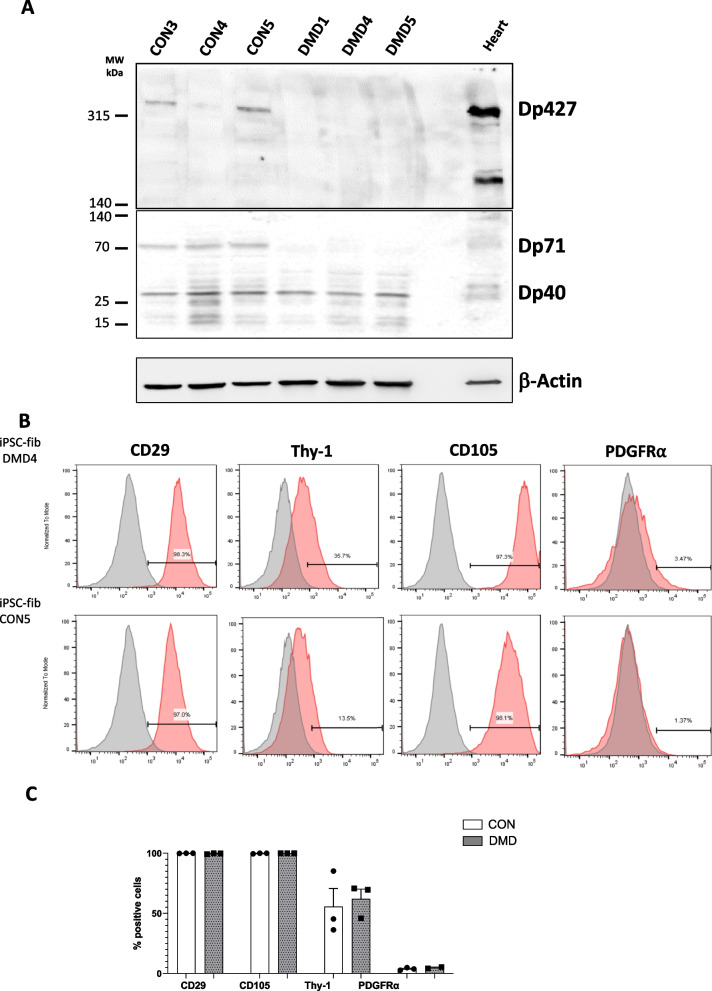
Characterization of iPSC-derived cardiac fibroblasts from DMD and control patients. **A**: Western blot analysis of dystrophin protein expression in human iPSC-derived cardiac fibroblasts. 80 µg of iPSC-derived cardiac fibroblasts and 10 µg of heart were loaded, transferred on membrane and revealed with monoclonal antibody against the C-terminal end of the protein. Three major bands at 427 kDa, 71 kDa and 40 kDa were observed in control patients whereas DMD patients expressed only the 40 kDa and faint amount of 71 kDa. **B**: Representative Flow cytometry graphs obtained with iPSC-derived cardiac fibroblasts generated from DMD and control patients. Cells were first identified on a forward scatter/side scatter (FSC-A/SSC-A) dot plot, doublets were removedand live cells were selected. Cells expressed different levels of CD29, CD105, CD90 (thy1.1) and PDGFRα. Endothelial and leucocyte markers, CD31 and CD45 respectively, were negative (not shown). **C**: Flow cytometry quantification of expressed markers Data are presented as mean ± SEM

Western blot analysis revealed that a 427KDa form of dystrophin, recognized in control hiPSC-fibs was absent in all DMD hiPSC-fibs patients. The 71 kDa isoform was expressed control hiPSC-fibs whereas a faint signal was observed in DMD hiPSC-fibs. In contrast the 42 kDa isoform was present in both control and DMD hiPSC-fibs (Fig. 1A). Similar results were obtained with affinity purified polyclonal antibodies (Additional file 1: Fig. S1). To explore the consequences of dystrophin deletion on the hiPSC-fibroblasts gene expression and phenotype, we first analyzed fibroblast markers expressed by control and DMD hiPSC-fibs by flow cytometry. All patients' fibroblasts exhibited a fibroblast-like morphology and were negative for CD31 and CD45, regardless of genotype (Data not shown). The control and DMD hiPSC-fibs were all positive for CD29 and CD105 whereas about 60 ± 10% expressed Thy1.1, and 4 ± 1% expressed PDGFRα with no significant differences between the genotypes (Fig. 1B–C).

### RNA expression analysis revealed altered metabolic pathways in DMD hiPSC-fibroblasts

**Fig. 2:**
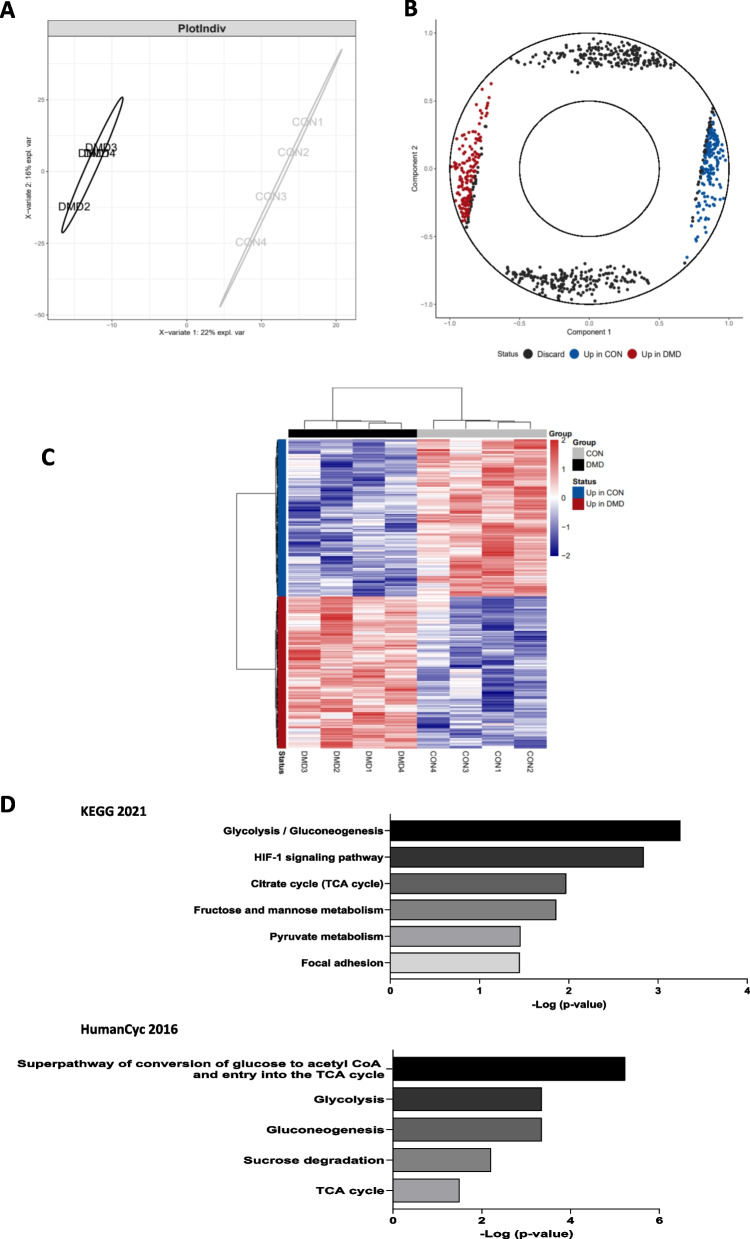
Transcriptome analysis of iPSC-derived cardiac fibroblasts from DMD and control patients. sPLS-DA was used to select genes that discriminate between DMD (black) and CON (grey) samples. iPS derived cardiac fibroblasts were obtained from 3 DMD patients and 3 healthy donors. One control and one DMD were run in duplicate. **A**: Individual plot shows that component 1 (x-axis) captured the variance between groups and that component 2 (y-axis) consisted mainly of inter-group variation. **B**: Variable (gene) plot of selected genes (keepX set to 400 per component) either up in DMD (red) or up in CON (blue). The x and y axes correspond to the sample placement of Fig. 2A. Black points were discarded either because the y-position was more significant than the x-position or if that gene had a vip-score of < 1. **C**: Heatmap of differentially expressed genes selected from Fig. 2B.The row annotation colors are as in Fig. 2B (Up in DMD (red) and Up in CON (blue)). The column annotation is colored as in Fig. 2A. Normalized RNA expression is row-scaled so that red and blue indicate expression above or below the row mean. EnrichR was used to find significantly enriched pathways with the DMD up-regulated genes, as selected by sPLS-DA, using both **D**: Kyoto encyclopedia of genes and genomesdatabase (KEGG2021)as well as the **E**: human metabolic pathways database (HumanCyc 2016). Bar length is indicative of significance (− Log10(*P*.Value)). Principal enriched pathways include TCA cycle metabolism, gluconeogenesisand glycolysis

To select gene expression altered by the loss of dystrophin expression in DMD and control hiPSC-fibs, we used sPLS-DA (keeping 400 variables on 2 components) from mixOmics in order to select genes with the most importance in separating samples based on DMD/CON (Component 1) as seen in the individual (sample) plot show in Fig. 2A (left panel), with DMD samples in black and CON samples in light grey. The results of the mixOmics vip program was used to further select the best genes by filtering with vip scores for component 1 above 1.0 and requiring that the x position was greater than the y position within the variable (gene) plot shown in Fig. 2A (right panel). The final set of genes selected as upregulated in DMD (red) or CON (blue) samples were then clustered with ward.D clustering of the Euclidean distance in a heatmap (Fig. 2B, same red/blue coloring as in Fig. 2A on rows and black/grey coloring on columns). The heatmap is showing a total of 323 genes, 159 up in DMD hiPSC-fibs patients and 164 up in CON hiPSC-fibs patients. The 159 up in DMD genes were used with EnrichR to select significantly enriched pathways up-regulated in DMD fibroblasts. The KEGG (Fig. 2C) and HumanCyc (Fig. 2D) databases revealed many common DMD up-regulated pathways related to glycolysis, gluconeogenesis and the TCA cycle (Additional file 1: Fig. S2). In addition, these results suggest DMD fibroblasts may be suffering hypoxic stress as we found genes to be upregulated from the HIF-1 signaling pathway.

### Loss of dystrophin in DMD hiPSC-fibs enhanced glycolysis

**Fig. 3:**
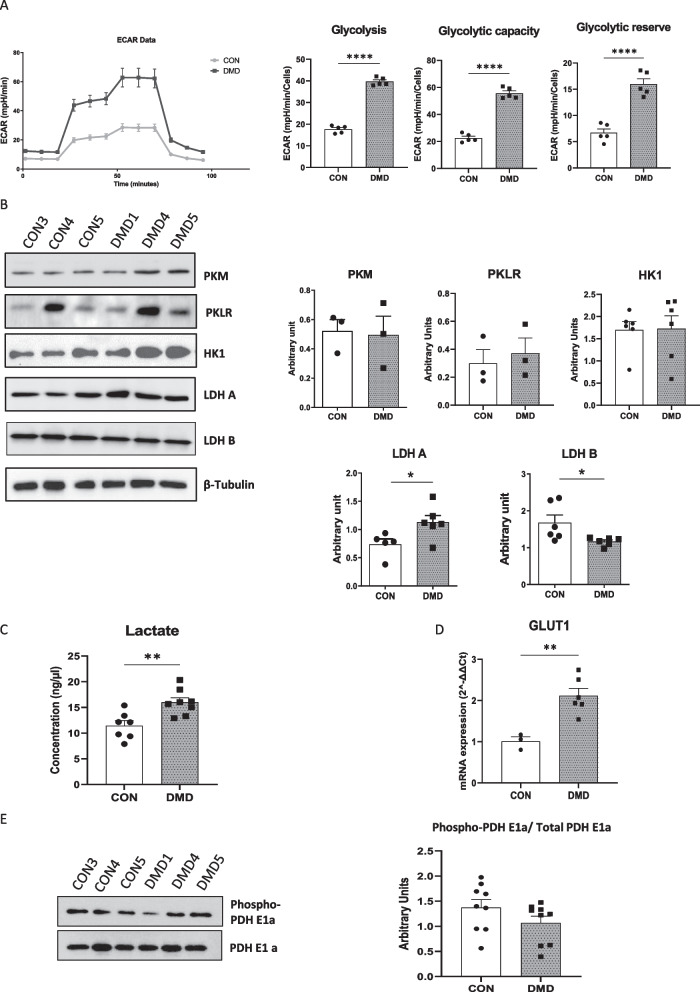
iPSC-derived cardiac fibroblasts from DMD patients exhibit high glycolytic capacity. **A**: The extracellular acidification rate (ECAR) profile of iPSC-derived cardiac fibroblasts subjected to a glycolysis stress test and the individual parameters for glycolysis, glycolytic capacityand glycolytic reserve. iPSC-derived cardiac fibroblasts were seeded to 80,000 cells/well and incubated for 24 h. ECAR was measured under basal conditions followed by the sequential addition of 10 mM glucose, 0.5 µM oligomycin and 100 mM 2-deoxyglucose (2-DG). Each data point represents an ECAR measurement obtained from the six biological samples of different batches of iPSC-derived cardiac fibroblasts. Data are expressed as mean ± SEM***p < 0.001 by non parametric t test. **B**: The protein expression levels of pyruvate kinase (muscular isoform-PKM), pyruvate kinase (L/R isoform), lactate dehydrogenase A and B, were quantified in iPSC-derived cardiac fibroblasts by western blotting and normalized to beta tubulin. Data are expressed as mean ± SEM of replicates of the six biological samples. **C**: Quantification of Intracellular l-lactate in iPSC-derived cardiac fibroblasts. Data are presented in mean ± SEM of replicates of the six biological samples, performed independently on different differentiation batches. ***p < 0.001 by non parametric *t* test. **D**: RT-PCR analysis of glucose transporter 1 mRNA expression levels. **p < 0.01 by non parametric t-test. **E**: Western blot analysis of phosphorylation on serine 293 of the PDH-E1α subunit in iPSC-derived cardiac fibroblasts.Data are presented in mean ± SEM of replicates of the six biological samples, performed independently on different differentiation batches

To further explore metabolic modifications, we first analyzed glycolysis in control and DMD hiPSC-fibs using the glycolysis stress test on the Seahorse analyser. DMD hiPSC-fibs showed up-regulated extracellular acidification, glycolytic rate, and glycolytic capacity compared to n control hiPSC-fibs, indicating an important up-regulation of glycolysis in the absence of dystrophin isoforms (Fig. 3A).

Then, the expression of the principal enzymes involved in glycolysis was analyzed by immunoblot. Hexokinases are the initial enzyme of the glycolytic pathway, catalyzing the phosphorylation of glucose by ATP to glucose-6-P. Immunoblots revealed some heterogeneity within both DMD and control hiPSC-fibs, however no significant changes in hexokinase I, the main isoform expressed in hiPSC-fibs was observed in DMD as compared to control hiPSC-fibs. Similarly the pyruvate kinase isoforms (PKM and PKLR) that produce pyruvate from its substrate phosphoenolpyruvate were not differentially expressed in DMD or control hiPSC-fibs (Fig. 3B). However, a light up-regulation of lactate dehydrogenase A and a down regulation of lactate dehydrogenase B were observed in DMD hiPSC-fibs as compared to control hiPSC-fibs (Fig. 3B). While LDH isoform B (LDHB) catalyzes the transformation of lactate into pyruvate, LDH-A catalyzes the inverse reaction. Under poor oxidative phosphorylation situations, the fermentation of pyruvate to lactate by LDH-A, regenerates NAD^+^ from NADH in order to maintain glycolysis. The enhanced capacity of DMD hiPSC-fibs to produce lactate was further confirmed by the increase in cellular lactate concentration (Fig. 3C). The prevalence of the glycolytic pathway in DMD hiPSC-fibs was further supported by an increased expression of glucose transporter 1 (GLUT-1), emphasizing a preponderant use of glucose for cellular metabolism in the DMD hiPSC-fibs as compared to control hiPSC-fibs (Fig. 3D).

### DMD hiPSC-fibs showed reduced mitochondrial respiration

**Fig. 4:**
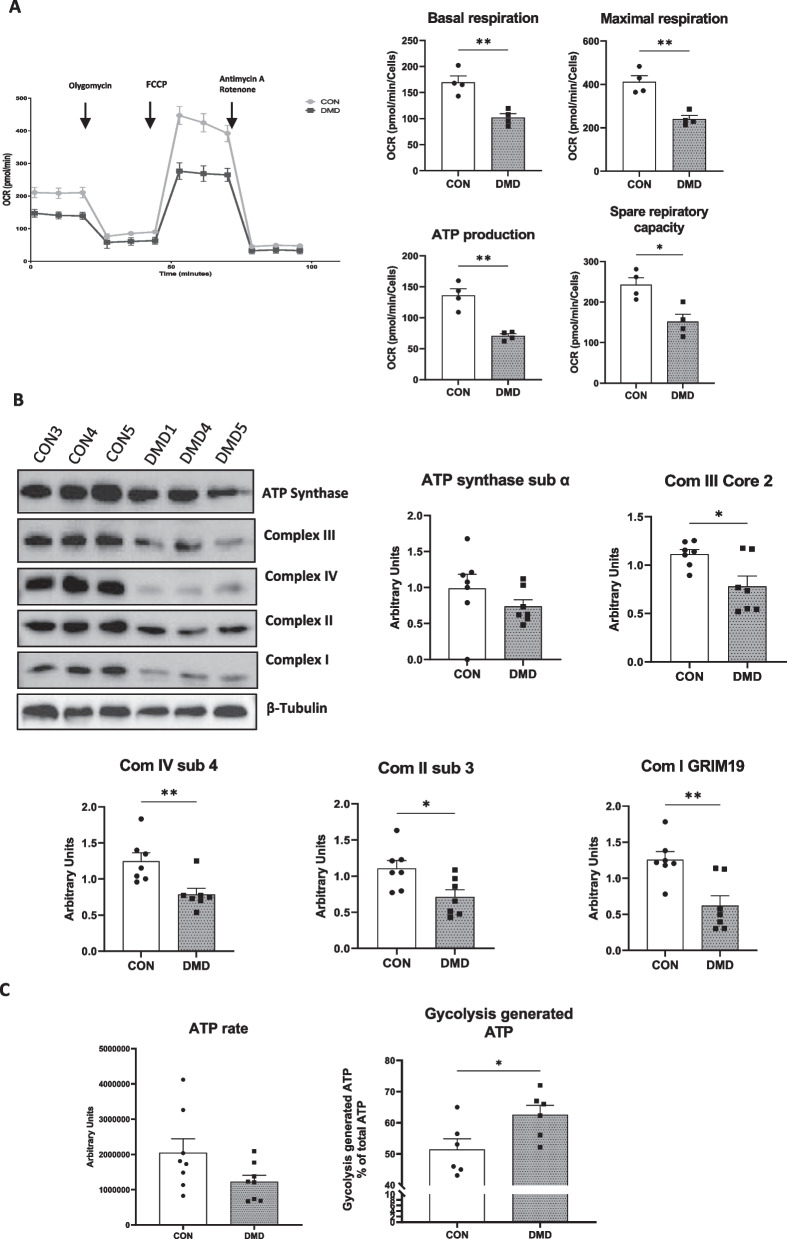
Dystrophin loss impairs mitochondrial bioenergetics. **A**: Mitochondrial respiration of iPSC-derived cardiac fibroblasts was measured in three different DMD patients and three control patients. Mitochondrial stress test shows the OCR trace measured sequentially under basal conditions, following inhibition of ATP synthase (with oligomycin), uncoupling the electron transporter chain (ETC) with FCCP and blocking complex I and III with rotenone and antimycin A (Left graph). Basal respiration, maximal respiration, ATP production and spared respiratory capacity are reported in the left panels. Three independent experiments were performed. Non parametric t-test was applied to identify statistically significant differences between DMD and control. *p < 0.05, **p < 0.01. **B**: Western blot of respiratory complex subunits (OXPHOS) and quantification of experimental replicates of the six biological samples obtained from multiple differentiation batches**.** Non parametric t-test was applied to determine the statistically significant differences between DMD and control. *p < 0.05, **p < 0.01.**C**: Quantification of ATP levels in iPSC-derived cardiac fibroblasts from DMD and control patients (left panel). Cells were incubated for 60 min with sodium iodoacetate, a glycolysis inhibitor, to assess glycolysis-dependent ATP production (right panel). Two technical replicates from each of the three biological samples were performed independently. Non parametric t-test was applied to determine the statistically significant differences between DMD and control. *p < 0.05

The up-regulated lactate production observed in DMD hiPSC-fibs may reflect altered mitochondria function and/or impaired glycolytic pyruvate to acetyl coA conversion depleting the mitochondrial supply of with energetic substrates. Thus, we assessed the expression of the pyruvate dehydrogenase complex and the phosphorylation of the PDHE1a subunit, which reduces the enzymatic activity of the complex. The DMD hiPSC-fibs showed decreased phosphorylation of the PDH E1a suggesting a more efficient ability to decarboxylate pyruvate into acetyl-CoA and, thereby feeding the TCA cycle and producing energy by oxidative phosphorylation (OXPHOS)) (Fig. 3E). We next evaluated mitochondrial respiration of DMD hiPSC-fibs using the cell mito stress test on the Seahorse analyzer. A down-regulation of mitochondrial respiration was observed in DMDs, as evidenced by a decrease in the basal and maximal respiration, a lower spare respiratory reserve and mitochondrial ATP production (Fig. 4A). As mitochondrial respiration was reduced in DMD hiPSC-fibs, we determined the protein expression for components of the mitochondrial electron transport chain (ETC). Concordantly, the protein abundance of complex I subunit (NDUFB8), complex II Subunit (SDHB), complex III subunit (UQCRC2), complex IV (COXIV) and ATPase Synthase (ATP5A) were significantly diminished in DMD hiPSC-fibs (Fig. 4 B). We next assessed the cellular ATP levels in DMD and control hiPSC-fibs in a luciferase-based luminescence reaction. Despite the drastically reduced capacity to generate ATP by oxidative phosphorylation, the total cellular ATP level trended to decrease in DMD hiPSC-fibs as compared to controls (Fig. 4C). However, ATP derived from glycolysis was significantly up-regulated in DMD hiPSC-fibs, further strengthening the increased use of glycolysis in DMD hiPSC-fibs (Fig. 4C).

All together, these results indicate that the loss of dystrophin in hiPSC-fibs mitigates mitochondrial respiration and induced a metabolic switch toward glycolysis.

### DMD hiPSC-fibs exhibited lower expression of mitochondrial complex and mitochondrial number

**Fig. 5:**
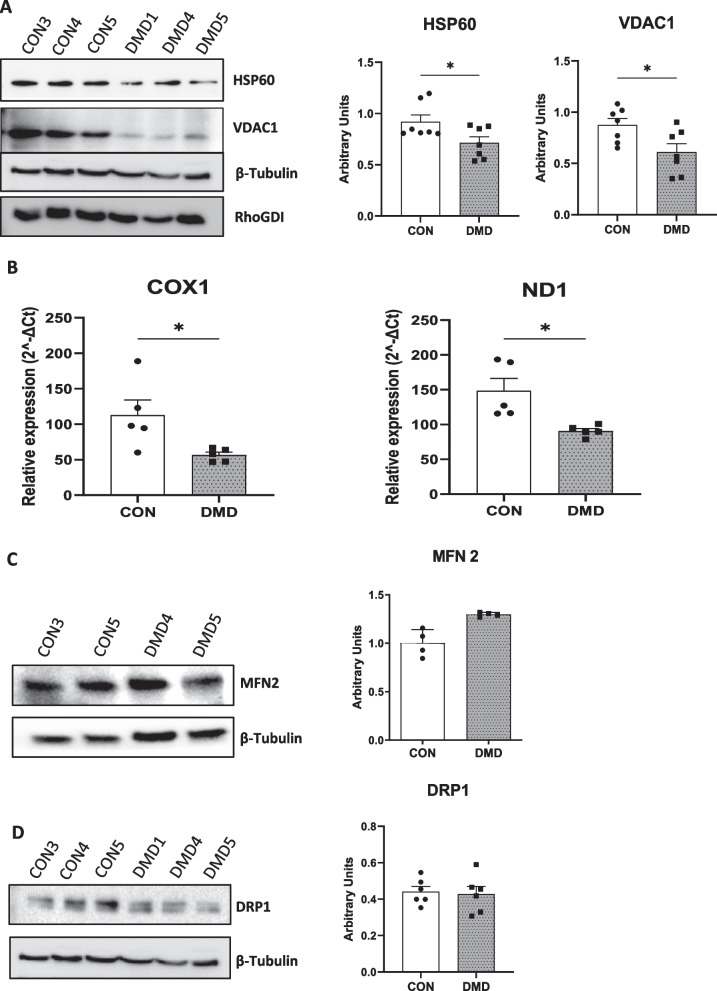
iPSC-derived cardiac fibroblasts from DMD patients showed a reduced number of mitochondria. **A**: Western blot (left) and quantification(right) of HSP60 and VDAC1 protein expression in iPSC-derived cardiac fibroblasts. Quantification was performed on six biological samples obtained from multiple differentiation batches. Non parametric t-test was applied to determine the statistically significant differences between DMD and control. *p < 0.05, **p < 0.01. **B**: The relative abundance of mtDNA genes COX1 and ND1 using nuclear gene β globulin as a reference, was measured by PCR in hiPSC-fibroblasts derived from control and DMD patients**.** Non parametric t-test was applied to determine the statistically significant differences between DMD and control. *p < 0.05. Western blot quantification of MFN2 (**C**) and DRP1 (**D**) protein expression in iPSC-derived cardiac fibroblasts. Non parametric t-test was applied to determine the statistically significant differences between DMD and control

We next determined whether the decreased expression of enzymes in the electron transport chain resulted from a reduction in the number of mitochondria in DMD. We first analyzed the expression of Heat shock protein 60 (HSP60), a mitochondrial chaperone and Voltage-Dependent Anion Channel (VDAC1), also used as a mitochondrial protein marker. Although some heterogeneity was observed within the DMD hiPSC-fibs, a significant decreased in HSP60 and VDAC1 expression was observed in the DMD hiPSC-fibs as compared to control hiPSC-fibs (Fig. 5A). In order to more precisely determine the mitochondria content within hiPSC-fibs, mitochondrial DNA to nuclear DNA ratio (mtDNA/nDNA) was assessed. The median content of mitochondrial genes COX1 and ND1 was reduced by about two-fold in DMD hiPSC-fibs, as compared to hiPSC-fibs obtained from control subjects (for mitochondrial COX1/ nuclear B2 microglobulin; 56 ± 4 in DMD vs 112 ± 21 in control and for mitochondrial ND1/ nuclear B2 microglobulin;90 ± 3 in DMD vs 148 ± 17 in control, p < 0.05 by Mann Whitney test) (Fig. 5B).

The reduced number of mitochondria observed in DMD could result from altered mitochondrial dynamics, which control the quality of mitochondria and maintain mitochondrial function. The molecular mechanisms underlying these processes rely on fusion (mitofusin 1 and 2) and fission proteins (dynamin-related protein), Western blot analysis revealed no significant modifications in the protein expression of DRP1 and mitofusin2 between DMD and control hiPSC-fibs (Fig. 5C–D).

These results showed a reduced mitochondrial content in DMD hiPSC-fibs that did not appear to be associated with impaired mitochondrial fusion or fission.

### DMD hiPSC-fibs exhibited spatial mitochondrial rearrangements and modification in actin microfilament number

**Fig. 6:**
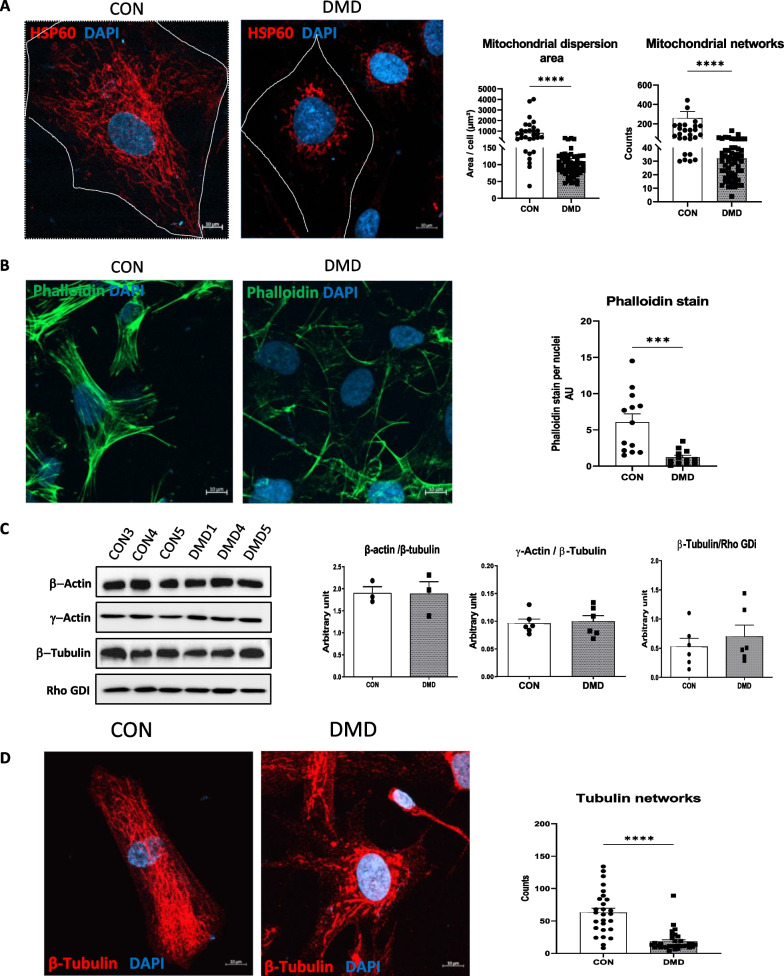
iPSC-derived cardiac fibroblasts from DMD patients displayed abnormal microfilament morphology and mitochondrial network. **A**: Representative 63 × confocal images of hiPSC-fibroblasts stained with anti-HSP 60 antibodyto show the mitochondrial network in iPSC-derived cardiac fibroblasts generated from control and DMD patients. The dashed white lines show plasma membrane of the cells. Quantification of mitochondrial network using MiNA(Mitochondrial Network Analysis) workflow within Fiji, for the ‘’mitochondrial footprint’’ or mitochondrial dispersion area (left) and the mitochondrial “networks’’(right). A t-test was applied to determine the statistically significant differences between DMD and control. ****p < 0.0001. **B**: Representative 63 × confocal images of phalloidin-stained filamentous actin and subsequent quantification. **C**: Western blot and quantification of beta actin, gamma actin, b tubulin and Rho GDI protein expression in iPSC-derived cardiac fibroblasts. Data are presented as mean ± SEM from analyses obtained from different batches of cardiac fibroblasts differentiated from iPSC.Non parametric t-test was applied to determine the statistically significant differences between DMD and control. ***p < 0.001. **D**: Representative 63 × confocal images of hiPSC-fibroblasts stained with anti β-tubulin antibody showing the microtubule network in iPSC-derived cardiac fibroblasts generated from control and DMD patients

We performed immunofluorescent staining with the anti-mitochondrial antibody hsp60 to assess the mitochondrial network. In contrast to control hiPSC-fibs, which showed mitochondrial distribution throughout the cytoplasm of the cell, the mitochondria of DMD hiPSC-fibs were mainly clustered around the nucleus of the cell with poor dispersion throughout the cytosol (Fig. 6A). The altered intracellular distribution of mitochondria in DMD hiPSC-fibs was characterized by a significantly reduced branching and elongation of the mitochondrial network (Fig. 6A). As mitochondria utilize the cytoskeletal network to move throughout the cell, we stained and visualized filamentous actin with Phalloidin. DMD hiPSC-fibs revealed a modified organization of actin filaments, with a reduced number and randomly oriented filament bundles as compared to control hiPSC-fibs, which showed well organized bundles (Fig. 6B). This loss of filamentous actin assembly and coherency was independent of β-actin and γ-actin protein content that appeared similar in both DMD and control hiPSC-fibs (Fig. 6C). Similarly, β -tubulin protein expression, a component of microtubules, was comparable in DMD and control hiPSC-fibs (Fig. 6C). However, microtubule structure in DMD hiPSC-fibs was slightly thicker than in control hiPSC-fibs, suggesting that F-actin disorganization in DMD interferes with α- and β-tubulin assembly (Fig. 6C).

All together these results showed that dystrophin plays an indispensable role in bundling filamentous actin and mitochondria distribution within the cell.

### DMD hiPSC-fibs exhibited myofibroblast phenotype and a high sensitivity to pro-fibotic stress

**Fig. 7:**
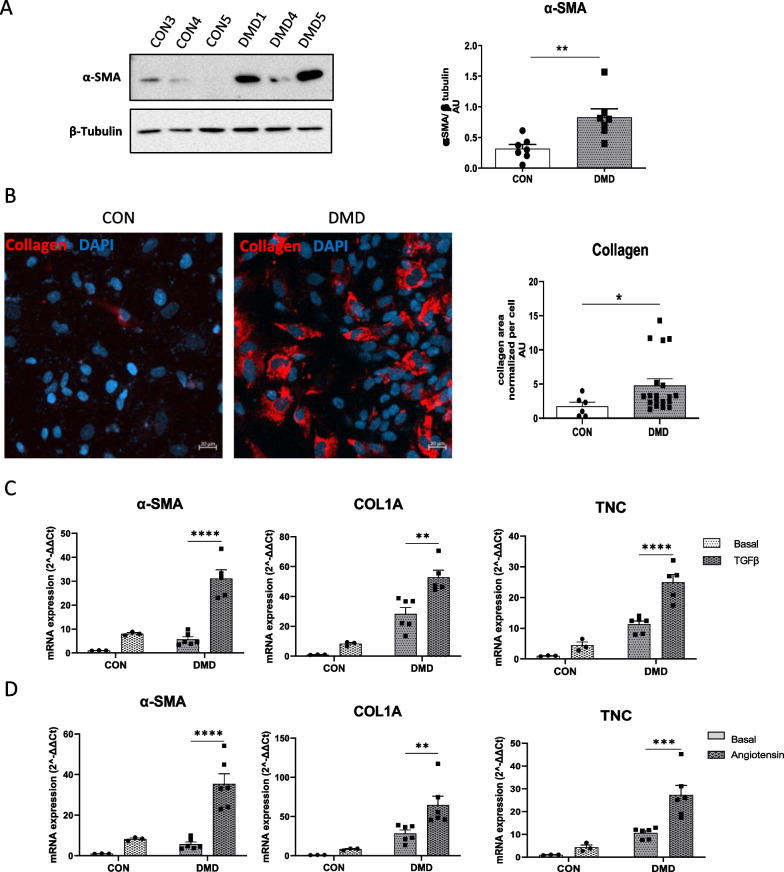
iPSC-derived cardiac fibroblasts from DMD patients display a myofibroblast phenotype and an increased sensitivity to stress. **A**: Representative immunoblot of α-sma expression obtained in iPSC-derived cardiac fibroblasts generated from DMD and control patients and quantification. Data are presented as mean ± SEM of the six biological samples. Replicates were performed independently on different differentiation batches. **B:** Immunofluorescence staining of collagen I in iPSC-derived cardiac fibroblasts generated from DMD and control patients and quantification of α-SMA, COL1A and TNC expression in iPSC derived cardiac fibroblast treated with 10 ng/ml TGFβ (**C**) or 100 ng/ml angiotensin II (**D**) for 24 h. One-way ANOVA was applied to determine statistically significant differences between the basal condition and TGFβ or angiotensin treatments

We further examined the consequences on cell mitochondrial and microfilament rearrangements in DMD hiPSC-fibs by investigating the expression of alpha-smooth muscle actin (α-SMA) and collagen 1 proteins used as markers of myofibroblasts. DMD hiPSC-fibs express higher levels of α -SMA protein compared to control hiPSC-fibs (Fig. 7A). In addition, more cells were positive for collagen 1, suggesting that loss of dystrophin was associated with a change in cellular phenotype (Fig. 7B). Then, we evaluated if the DMD hiPSC-fibs were more prone to differentiate into myofibroblasts under profibrotic stress, despite deficient actin cytoskeleton network. Treatment with the profibrotic factors TGF β or angiotensin II induced a higher expression of alpha-smooth muscle actin, *COL1A1* and tenascin C in DMD hiPSC-fibs than in control hiPSC-fibs (Fig. 7C–D). These results showed that the dystrophin plays a cell-intrinsic function in restraining fibroblast activation.

## Discussion

In this study, we revealed the expression of dystrophin isoforms in hiPSC derived cardiac fibroblasts. A 427 kDa dystrophin protein was only found in control patients and not in the DMD patients who have out of frame deletions of exons or a spontaneous point mutation that induce a frameshift and a premature stop signal. The lack of dystrophin in human iPSC derived fibroblasts triggers a cascade of noxious cellular effects leading to cytoskeletal remodeling, modified energy supply, myofibroblast phenotype and acute sensitivity to pro-fibrotic stress.

A previous study performed with the mdx mouse model of Duchenne Disease showed that microtubule disorganization and mitochondrial dysfunction occur concurrently in dystrophin-deficient muscle. However, the link between cytoskeletal disorganization and mitochondrial bioenergetics was not uncovered [24]. The modified actin cytoskeleton in DMD most likely results from the loss of the actin binding domains (ABD). Specific domains of dystrophin have been shown to interact with microtubules [25, 26], but the absence of dystrophin had little effect on microtubule structure, suggesting that dystrophin plays only a minor role in microtubule arrangement. In contrast, the disorganized F-actin, caused by the loss of dystrophin in cardiac fibroblasts appeared to muddle distribution of mitochondria within the cell, along with a reduction in mitochondria number. The organelle utilizes the cytoskeletal network to move throughout the cell, while microtubules are used for long-range transport, the actin network is used for short-range movements. Additionally, the correct positioning and docking of mitochondria at locations of nutrient abundance increases the efficiency of ATP generation [27]. The reshaped distribution and the reduced number of mitochondria in DMD hiPSC-derived cardiac fibroblasts blunted mitochondrial respiration, which was further confirmed by the lower expression of ETC complexes. A recent study reported impaired energy metabolism as well as abnormal mitochondrial structure and function in iPSC-derived cardiomyocytes generated from adult DMD patients, but not from a young DMD patient [28]. Similarly, mitochondrial function and biogenesis were found compromised in cardiomyocytes from the Duchenne mouse model mdx with “humanized” telomeres [29] suggesting that mitochondrial dysfunction accompanies dystrophin loss and may account for the etiology of dystrophic heart failure.

The use of glycolysis in strong preference to oxidative phosphorylation in human iPSC derived fibroblasts from DMD patients likely reflects a compensatory mechanism to counterbalance the dysfunctional mitochondria as global cell ATP production was maintained. The metabolic remodeling induced by the loss of dystrophin in DMDs may be the trigger for enhanced myofibroblast differentiation. Indeed, glycolysis was found to be a critical metabolic enabler of cardiac fibroblast activation whereas glucose oxidation alleviates it [30]. For example, inhibition of glycolysis blunted the differentiation of lung fibroblasts into myofibroblasts and attenuated profibrotic phenotypes in myofibroblasts isolated from the lungs of patients with idiopathic pulmonary fibrosis [31].The contribution of HIF-1a pathway in the metabolic switch observed in DMD hiPSC-derived cardiac fibroblasts was not examined in the present study and needs to be further explored as over-expression of hypoxia-inducible factor 1-α, was shown to potentiate myofibroblast differentiation [32].

In addition to be constitutively activated, DMD hiPSC-derived cardiac fibroblasts have an enhanced fibrotic response when stimulated with TGF-β or angiotensin II. Studies have shown that dysregulation of the normal wound healing response may also potentially lead to fibrosis. Indeed, depletion of activated cardiac fibroblasts by immunological targeting alleviates cardiac fibrosis and rescued cardiac contraction showing that cardiac fibroblasts are important regulator of heart physiopathology [33].Therefore, the accumulation of fibrotic tissue in the heart of DMD patients may not only be secondary to cardiomyocyte death, but results from inappropriate activation of fibroblasts.

Our study highlights the relationship between cytoskeletal dynamics, metabolism of the cell and myofibroblast differentiation. It provides a new mechanism by which inactivation of dystrophin in non-cardiomyocyte cells worsens cardiac fibrosis and leads to increased severity of cardiopathy.

Limitations concerning the isoforms expressed in control and DMD hiPSC-derived cardiac fibroblasts should be acknowledged in our study. Whereas the highest isoform of dystrophin protein was persistently found in control hiPSC, smaller isoforms such as 71 kDa or 40 kDa could be also observed in control and DMD hiPSC-fibs patients who have large deletions encompassing multiple exons or a spontaneous point mutation. The transcription of mRNAs encoding the 71 kDa (Dp71) and the 40 kDa (Dp40) isoforms of dystrophin are initiated from the same promoter in intron 62 [34]. Dp40 results from alternative splicing in the C terminal portion of the protein. The positive bands observed at 71 kDa and 40 kDa suggest that the expression of Dp71 and Dp40 isoforms might be ubiquitous, however fragments generated by proteolysis cannot be excluded.

### Supplementary Information

**Additional file 1**. For data supplemment figure 1: Western blot analysis of dystrophin protein expression in human iPSC-derived cardiac fibroblasts with affinity purified polyclonal antibodies (3 to 4 technical replicates were performed). Immunodetection of b-actin was used as loading control. A: Expression of the 427kDa protein was retrieved with polyclonal antibodies raised against aa 410-450, B: Expression of the 427kDa, the 71kDa and the 40kDa protein isoforms was retrieved with polyclonal antibodies raised against C-terminal end. For data supplemment figure 2: EnrichR was used to identify significantly up-regulated pathways in DMD hiPSC-fibs. List of top genes up regulated in DMD hiPSC-fibs. and associated with metabolic pathways from the KEGG and HumanCyc databases.

## Data Availability

All data used for the current study are available from the corresponding author (nathalie.pizzinat@inserm.fr) upon reasonable request. Raw data of RNA-seq are uploaded to GEO database with accession number GSE237014.
