# Correction: IPSC derived cardiac fibroblasts of DMD patients show compromised actin microfilaments, metabolic shift and pro-fibrotic phenotype

**DOI:** 10.1186/s13062-023-00417-2

**Published:** 2023-10-07

**Authors:** Salwa Soussi, Lesia Savchenko, Davide Rovina, Jason S. Iacovoni, Andrea Gottinger, Maxime Vialettes, Josè-Manuel Pioner, Andrea Farini, Sara Mallia, Martina Rabino, Giulio Pompilio, Angelo Parini, Olivier Lairez, Aoife Gowran, Nathalie Pizzinat

**Affiliations:** 1 National Institute of Health and Medical Research (INSERM), I2MC, U1297, Toulouse, France; 2 Unit of Vascular Biology and Regenerative Medicine, Centro Cardiologico Monzino IRCCS, Milan, Italy; 3 National Institute of Health and Medical Research (INSERM) U1297 I2MC, Bioinformatic Core Facility, I2MC, Toulouse, France; 4 Department of Biomedical, Surgical and Dental Sciences, Università Degli Studi di Milano, Milan, Italy; 5 Department of Biology, Univer sity of Florence, Florence, Italy; 6 Neurology Unit, Fondazione IRCCS Ca’ Granda Ospedale Maggiore Policlinico, Milan, Italy; 7 University Toulouse III, 118 route de Narbonne, 31062 Toulouse, CEDEX 9, Toulouse, France

**Biology Direct (2023) 18:41**

10.1186/s13062-023-00398-2

After publication of this article [1], it was brought to our attention that the author name should be corrected from Maxime Viallettes to Maxime Vialettes.

The original publication has been corrected.
