# Supplementary material for: IPSC derived cardiac fibroblasts of DMD patients show compromised actin microfilaments, metabolic shift and pro-fibrotic phenotype
Source: Biol Direct. 2023 Jul 27;18:41. doi: 10.1186/s13062-023-00398-2 (PMC10373315; doi:10.1186/s13062-023-00398-2)
Supplement: Supplementary file 1 — Additional file 1. For data supplemment figure 1: Western blot analysis of dystrophin protein expression in human iPSC-derived cardiac fibroblasts with affinity purified polyclonal antibodies (3 to 4 technical replicates were performed). Immunodetection of b-actin was used as loading control. A: Expression of the 427kDa protein was retrieved with polyclonal antibodies raised against aa 410-450, B: Expression of the 427kDa, the 71kDa and the 40kDa protein isoforms was retrieved with polyclonal antibodies raised against C-terminal end. For data supplemment figure 2: EnrichR was used to identify significantly up-regulated pathways in DMD hiPSC-fibs. List of top genes up regulated in DMD hiPSC-fibs. and associated with metabolic pathways from the KEGG and HumanCyc databases. [file 13062_2023_398_MOESM1_ESM.pdf]

Data supplement Figure 1

A

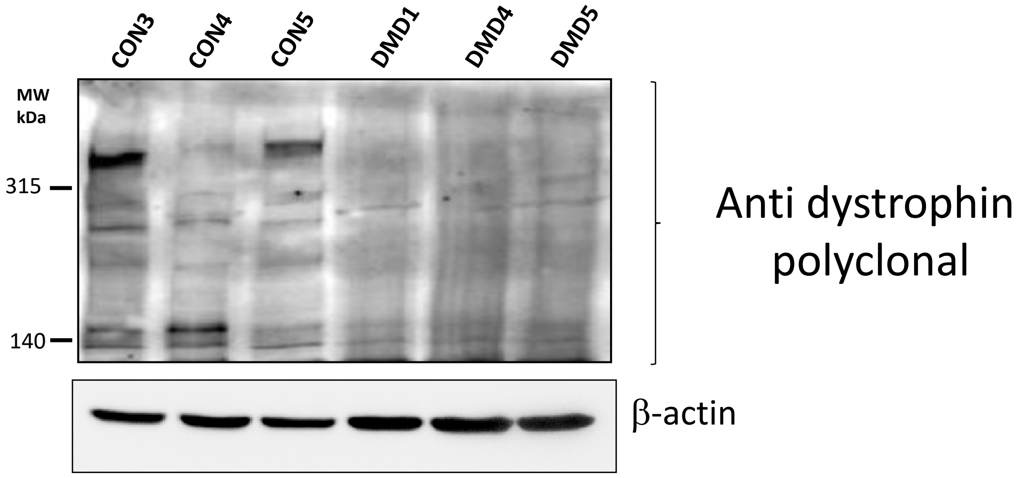

B

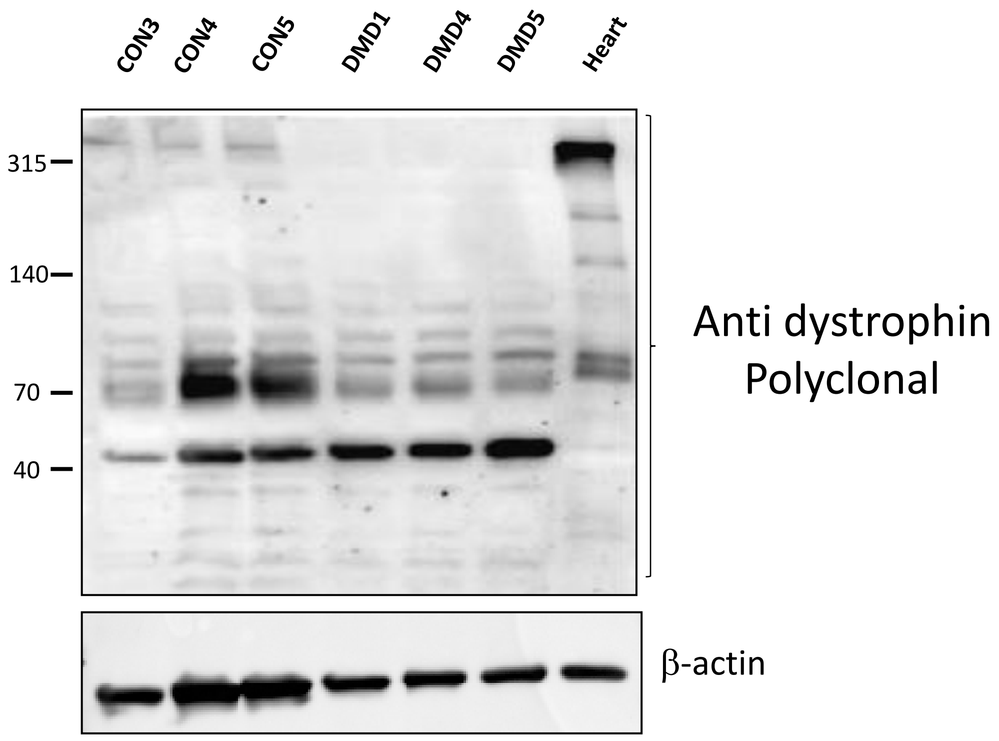

Western blot analysis of dystrophin protein expression in human iPSC-derived cardiac fibroblasts with affinity purified polyclonal antibodies (3 to 4 technical replicates were performed).

A: Expression of the 427kDa protein was retrieved with polyclonal antibodies raised against aa 410-450,

B: Expression of the 427kDa, the 71kDa and the 40kDa protein isoforms was retrieved with polyclonal antibodies raised against C-terminal end.

Data supplement Figure 2

	Up regulated genes in DMD						
	KEGG 2021 Human						
Rank	Term	P.Value	adj.P.Value	Z.Score	CombinedScore	Gois	Database
1	Glycolysis / Gluconeogenesis	0,00017186	0,013521495	10,63602151	92,20218799	LDHA,TPI1,PDHA1,PGK1,ALDOC	KEGG_2021_Human
2	HIF-1 signaling pathway	0,00020644	0,013521495	7,718251124	65,4934113	LDHA,PDHA1,PGK1,ALDOC,EIF4E,RBX1	KEGG_2021_Human
3	Nucleotide excision repair	0,00047759	0,02085459	12,19898352	93,28277567	POLE4,POLD2,GTF2H2,RBX1	KEGG_2021_Human
4	Ribosome	0,00146782	0,041648666	5,217149417	34,0365813	MRPL3,RPL36,RPL35,FAU,MRPL12,MRPL11	KEGG_2021_Human
5	Citrate cycle (TCA cycle)	0,00158964	0,041648666	14,48684211	93,356765	FH,PDHA1,SUCLG2	KEGG_2021_Human
6	Fructose and mannose metabolism	0,00210026	0,045855692	13,03618421	80,37712014	PFKFB4,TPI1,ALDOC	KEGG_2021_Human
7	RNA transport	0,0033236	0,062198845	4,399328859	25,10567646	EIF4A1,EIF1AX,PABPC4,GEMIN5,EIF4E,EIF3A	KEGG_2021_Human
8	Pyruvate metabolism	0,00576774	0,094446674	8,882027512	45,7910768	FH,LDHA,PDHA1	KEGG_2021_Human
	Human Cyc 2016						
Rank	Term	P.Value	adj.P.Value	Z.Score	CombinedScore	Gois	Database
1	superpathway of conversion of glucose to acetyl CoA and entry into the TCA cycle Homo sapiens PWY66-407	1,43E-06	1,43E-05	19,93791946	268,3809288	FH,TPI1,PDHA1,PGK1,SUCLG2,ALDOC	HumanCyc_2016
2	glycolysis Homo sapiens PWY66-400	0,00081989	0,00273298	18,63157895	132,4022519	TPI1,PGK1,ALDOC	HumanCyc_2016
3	gluconeogenesis Homo sapiens PWY66-399	0,00081989	0,00273298	18,63157895	132,4022519	TPI1,PGK1,ALDOC	HumanCyc_2016
4	sucrose degradation Homo sapiens PWY66-373	0,00162054	0,004051341	43,22222222	277,7026945	TPI1,ALDOC	HumanCyc_2016
5	TCA cycle Homo sapiens PWY66-398	0,0075193	0,015038607	17,28104575	84,50918235	FH,SUCLG2	HumanCyc_2016

List of top genes up regulated in DMD and associated with metabolic pathways from the KEGG and HumanCyc databases
